# Is insurance instability associated with hypertension outcomes and does this vary by race/ethnicity?

**DOI:** 10.1186/s12913-020-05095-8

**Published:** 2020-03-16

**Authors:** Nancy R. Kressin, Norma Terrin, Amresh D. Hanchate, Lori Lyn Price, Alejandro Moreno-Koehler, Amy LeClair, Jillian Suzukida, Sucharita Kher, Karen M. Freund

**Affiliations:** 1 VA Boston Healthcare System, Boston University School of Medicine, 801 Massachusetts Ave, Crosstown Center, Boston, MA 02118 USA; 2 Tufts Clinical and Translational Science Institute, Tufts University, 800 Washington St, Boston, MA 02111 USA; 3 Institute for Clinical Research and Health Policy Studies, Tufts Medical Center, 800 Washington St., Box #63, Boston, MA 02111 USA; 4 Division of Internal Medicine and Primary Care, Department of Medicine, Tufts Medical Center, 800 Washington St, Boston, MA 02111 USA; 5 Division of Pulmonary, Critical Care and Sleep Medicine, Department of Medicine, Tufts Medical Center, 800 Washington St, Boston, MA 02111 USA; 6 Institute for Clinical Research and Health Policy Studies, Division of Internal Medicine and Primary Care, Department of Medicine, Tufts Medical Center, 800 Washington St., Box #63, Boston, MA 02111 USA

**Keywords:** Insurance, Health, Health status disparity, Hypertension

## Abstract

**Background:**

Stable health insurance is often associated with better chronic disease care and outcomes. Racial/ethnic health disparities in outcomes are prevalent and may be associated with insurance instability, particularly in the context of health insurance reform.

**Methods:**

We examined whether insurance instability was associated with uncontrolled blood pressure (UBP) and whether this association varied by race/ethnicity. We used a retrospective longitudinal observational cohort study of patients diagnosed with hypertension who obtained care within two health systems in Massachusetts. We measured the UBP, insurance instability, and race of 43,785 adult primary care patients, age 21–64 with visits from 1/2005–12/2013.

**Results:**

We found higher rates of UBP for blacks and Hispanics at each time point over the entire 9 years. Insurance instability was associated with greater rates of UBP. Always uninsured black patients fared worst, while white and Hispanic patients with consistent public insurance fared best.

**Conclusions:**

Stable insurance of any type was associated with better hypertension control than no or unstable insurance.

## Background

Stable health insurance coverage is often associated with better chronic disease outcomes (e.g., hypertension) by ensuring consistent access to outpatient services including detection of disease, disease monitoring, and medications, [1–4]. The literature, however, has included conflicting findings. For example, some studies showed that insurance coverage is associated with better blood pressure (BP) outcomes, [2, 3] while others have not [5]. Conversely, insurance *instability*, or “churning” (switches in insurance coverage or gaps without coverage), has been associated with worse health or healthcare utilization outcomes, [1, 6] though prior examinations focused on self-reported health [6] or healthcare utilization outcomes, [1] rather than clinical outcomes.

Racial and ethnic health disparities in outcomes of chronic disease care are pervasive, well documented, and consistently linked to variations in health insurance coverage [7, 8]. Thus, insurance instability may contribute to disparities in outcomes of chronic disease care, similar to its known effects on delays in accessing diagnostic cancer care services [9].

Insurance reform may be associated with insurance instability as it fosters changes in insurance products and coverage. With the explicit goal of reducing disparities, Massachusetts (MA) Health Insurance Reform (2006–07) extended comprehensive health insurance coverage to 98% of the state’s residents, with disproportionately greater gains in coverage among racial/ethnic minorities and the poor [10]. Though MA had a robust safety net prior to this insurance reform, additional insurance products were made available to those individuals with Medicaid or whose incomes fell below 300% of the federal poverty line, further strengthening the safety net. The association of insurance instability with chronic disease outcomes, and the racial/ethnic disparities in such associations, have not been fully examined in this context.

### Objectives and hypothesis

Focusing on the highly prevalent chronic condition of hypertension, which has documented disparities in the outcome of BP control (and its sequelae, including strokes and target organ damage), [11–13] we used clinical data from two large healthcare systems in Massachusetts (an early adopter of health insurance reform) to examine whether insurance instability was associated with blood pressure outcomes and, if so, whether this association varied by patients’ race/ethnicity. We anticipated that greater insurance stability would be associated with better control of blood pressure, with differential associations based on race/ethnicity, hypothesizing more negative associations of instability with outcomes among Black, Hispanic and Asian patients.

## Methods

### Overview

Using electronic medical record (EMR) data from patients at two urban safety net clinical systems (hospitals plus affiliated community health centers), we conducted a retrospective longitudinal observational cohort study of patients with hypertension receiving primary care services during the time period spanning MA reform. We examined rates of uncontrolled BP (UBP) over time, by race/ethnicity, exploring whether UBP was differentially associated with insurance instability during the same time period. We chose to examine the concurrent association of these variables because the effect of antihypertensive medications on blood pressure values is short term, without a lag of months or even days. Given the potential association between switches in insurance coverage and associated changes in pharmacy formularies, provider networks, co-pays and deductibles on use of medications, we determined that a lagged analysis would be insensitive to these associations. Therefore we looked at BP measurements during the concurrent time frame as the instability in insurance coverage.

### Data sources

**(1) EMR.** Clinical and sociodemographic data were obtained from the EMR for adult primary care patients age 21–64 from the two care systems (including diagnoses, sex, race/ethnicity, use and location of care, insurance coverage at each visit, comorbidities, and BP measurements). **(2) Census tract information.** As the EMR did not include data on education or income, we obtained US Census tract-level sociodemographic data to estimate patients’ education level and income by geographic area. We used a web-based geocoding service [14] to assign geographic (census tract) area levels of education and income, based on the patients’ self-reported addresses from the hospital administrative data (7% missing due to inadequate addresses).

### Data time periods

We included data from January 2005 through December 2013, starting 18 months prior to implementation of Massachusetts (MA) Health Insurance Reform, including the 18-month transition window for implementation of the components of insurance reform, and continuing for 6 years after MA reform but before implementation of the national Affordable Care Act. Given that we were interested in examining the association of insurance instability with uncontrolled blood pressure in the context of MA health reform, we felt it important to include data from the pre-reform period as well as during the reform’s implementation and the time period subsequent to its implementation.

### Data organization

The primary outcome of interest was an assessment of an individual’s BP control within a specific time period (a “person-interval”). BP outcomes were defined per person-6-month interval (fixed time intervals for January – June and July – December for each year and person in the data set, such as 1/1/05–6/30/05 for patient #1). Membership in insurance instability groups (process and groups described below) was assigned for each person-interval.

### Subject inclusion criteria

We included data on patients aged 21–64 at the beginning of each person-interval, excluding patients once they reached 65 years, as Medicare eligibility ensures insurance stability, without risk of loss of eligibility. Then, to ensure inclusion of patients who were engaged with primary care for their hypertension (and whose BP outcomes could then be affected by care), we identified person-intervals from 1/1/05–12/31/13 where a diagnosis of hypertension was present, either in the problem list or as a billing code, during that interval or a preceding one. Patients became eligible for inclusion when they first satisfied this criterion and were allowed to remain in the dataset in subsequent years with any evidence of care within the health care system. Ninety percent of patients had more than one visit. Person-intervals without a PCP visit were excluded from analysis.

We dropped *individual patients* from the data set altogether if race/ethnicity group was always missing (7.6%) or identified as “other” (4.0%). *Person-intervals* were also dropped in the adjusted analyses if a covariate was always missing for the subject; this primarily applied to the Census tract variable, as some addresses could not be assigned to a Census tract (7%).

### Main outcome measure

#### Blood pressure outcome

We defined uncontrolled blood pressure based on Joint National Commission [9] (JNC7) standards applicable at the time [15]. UBP occurred when Systolic BP was greater than or equal to 140 mmHG or Diastolic BP was greater than or equal to 90 mmHg. As per the guidelines, for patients with Diabetes or Chronic Kidney Disease, these thresholds were lowered to 130 mmHg and 80 mmHg respectively (JNC7). When patients had multiple visits with BP measurements within the 6 month person-interval, we based our determination of BP control during that interval on the majority of values during the interval (e.g., BP was considered uncontrolled for the interval if uncontrolled at > 50% of visits). When exactly half of the visits indicated controlled BP, we considered BP as controlled. Moreover, if the current interval did not contain any BP measurements, we took the BP control status from the prior 6 month interval and carried it forward. If neither the current nor prior interval contained any BP recordings, but the interval 12 months prior to that did, then we assigned the current interval an outcome of uncontrolled BP. Given that half the person-intervals evidenced uncontrolled BP, and that reducing BP requires active clinical management, we posited that this was the most conservative approach. Finally, if all three were missing, we censored the interval. BP measurements were missing from 2% of the person-intervals.

### Independent and adjuster variables

#### Concurrent insurance instability groups

Using insurance billing information from each eligible visit, we examined patterns of insurance coverage over each person-interval. An insurance switch was defined as any switch among these five primary insurance coverage categories: private, Medicare, Medicaid, Commonwealth Care (the subsidized insurance option in MA developed as part of insurance reform for those up to 300% federal poverty level), and uninsured. From these, we created six categories of insurance instability, all defined at the level of the person-6-month-interval: 1) stable private insurance, 2) stable public insurance (always Medicare or always Medicaid or always Commonwealth Care), 3) those with insurance switches who *neither* gained *nor* lost insurance, 4) those with insurance switches who gained and did *not* lose insurance, 5) those with insurance loss, and 6) always uninsured.

#### Demographics

Using EMR information, we determined each patient’s age in years, sex, and race/ethnicity. We used Census tract data to determine area-level education (percent in the tract with high school (HS) graduation or greater) and income (median household tract income, in 2014 dollars).

Comorbidity burden was measured with the Charlson index, [16] calculated from diagnoses in the EMR problem list, and updated with new diagnoses as they became available over time.

### Statistical analyses

First, to examine patterns of blood pressure control by race/ethnicity over time, we plotted adjusted trajectories over time of the proportion of patients with UBP, by race/ethnicity. The analysis for estimating the trajectories used generalized estimating equations (GEE [17]; proc. GENMOD within SAS version 9.4) to account for correlated within-subject measurements. The model outcome was uncontrolled blood pressure (UBP, yes/no within a 6-month interval). The independent variables of interest were race/ethnicity and time. For model adjustment we included age as a categorical variable in 5-year increments; Charlson comorbidity score coded as no comorbidities, 1 comorbidity, or more than 1 comorbidity; percent in the census tract with at least a high school education; median income in the census tract; and site of care (3 categories: healthcare system A, its affiliated health centers, healthcare system B). The model used the logit link and autoregressive order 1 (AR [1]) correlation structure. Time was modeled as piece-wise linear, with 6 line segments, each covering 1.5 years. To test the interaction between race/ethnicity and time, we performed a global test of the 18 interaction terms (6 time variables for the piecewise model × 3 indicator variables for the 4-level race/ethnicity variable = 18 terms). The model was used to generate a prediction for each person every 6 months, and the means were plotted by race/ethnicity and 6-month interval.

To estimate the association of UBP with insurance instability, we used GEE with logit link and AR [1] correlation structure. The outcome was UBP and the independent variable of interest was the 6-category insurance instability variable. The adjusters were the same as for the previous model, except that we also adjusted for time as a categorical variable in increments of 6 months. Finally, we tested the interaction between insurance instability and race/ethnicity by performing a global test of the 15 interaction terms (5 indicator variables for the 6-level insurance instability variable × 3 indicator variables for the 4-level race/ethnicity variable = 15 terms).

## Results

### Characteristics of the patient samples

The sample was comprised of 43,785 patients (comprising 282,417 person-intervals; Table 1). The average age was 47.4 years (SD 10.9), and the race/ethnicity composition of the sample was 29% Non-Hispanic white (hereafter, “white”), 58% Non-Hispanic Black (hereafter, “black”), 6% Asian, and 7% Hispanic. The mean of the percentage of residents with high school graduation or higher in the subject’s Census tract was 82.3% (SD 10.3). The mean of the median income in the subject’s Census tract was $57,606 (SD $25,571). Fifty-four percent of patients came from healthcare system A, 37% from its affiliated health centers, and 9% from healthcare system B.

**Table 1:** Characteristics of the Sample in Two Massachusetts Urban Safety Net Hospital Systems, 2005–2013

Variable	***n*** = 43,785
Age (years) (mean (SD))	47.4 (10.9)
Male	20,178 (46.1)
Race	
Non-Hispanic White	12,520 (28.6)
Hispanic	3220 (7.4)
Black/African-American	25,221 (57.6)
Asian/Asian Pacific Islander	2824 (6.4)
Charlson Comorbidity Score	
0	23,764 (54.3)
1	11,155 (25.5)
≥ 2	8866 (20.2)
Percent in Census Tract High School Graduation or Higher (mean (SD))	82.3 (10.3)
Median Census Tract Income (2014 dollars, mean (SD))	$57,606 ($25,571)
Location	
Healthcare System A	23,454 (53.6)
Healthcare System A Affiliated Health Centers	16,180 (37.0)
Healthcare System B	4151 (9.4)

Note: Results are frequency (%) unless otherwise indicated. In the main sample, sex was missing for 1 person, and census tract education and income were missing for 3195 people (7%)

### Insurance instability and uncontrolled BP by race/ethnicity, over time

**Fig. 1:**
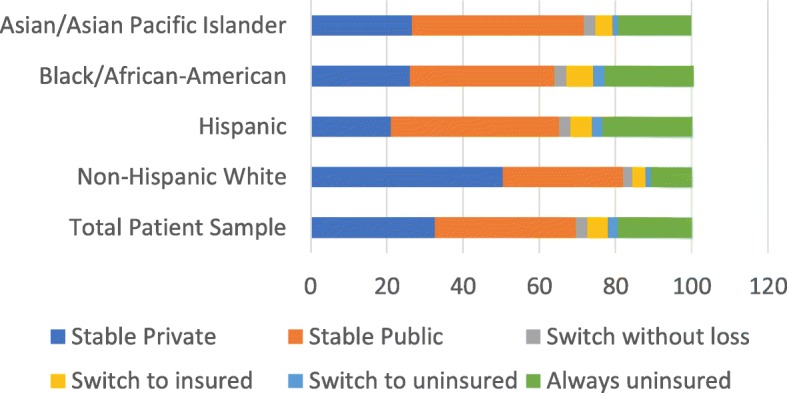
Insurance instability by race/ethnicity

**Fig. 2:**
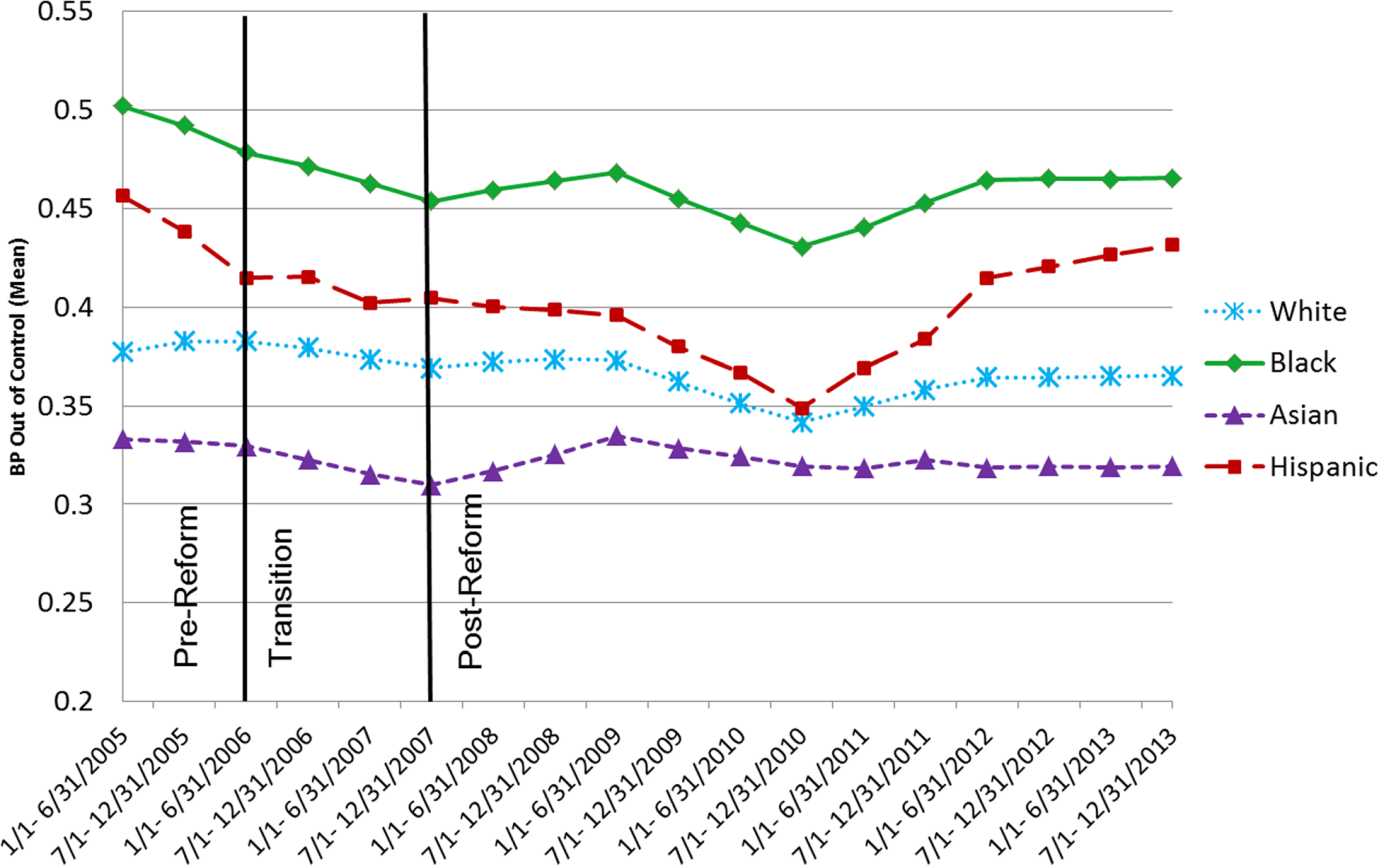
UBP by race and time

Insurance instability varied by race (Fig. 1). Rates of UBP were consistently different by race/ethnicity at each point in time (Fig. 2, p for race: < 0.001), with higher rates of UBP for blacks, followed by Hispanics, whites and Asians. The interaction between race and time was not statistically significant, implying no evidence of reduction in disparities over time.

Insurance stability was significantly (*p* < 0.001) and positively associated with better BP outcomes (Table 2). Having stable public insurance was associated with lower rates of UBP, as compared with stable private insurance. Both losing or gaining insurance and always being uninsured in a 6-month interval were associated with increased odds of UBP, compared with those with stable private insurance.

**Table 2:** Odds Ratios of Uncontrolled BP for Different Types of Insurance Stability/Instability, Compared to Stable Private Insurance, in Two Massachusetts Urban Safety Net Hospital Systems

Adjusted Odds Ratio of Uncontrolled BP		
Insurance Instability Group	n (6-month Person-Intervals)	OR (95% CI) ***p***-value
Stable Private	97,202	Reference
Stable Public	113,142	**0.93** (0.90, 0.96)
Switch without losing insurance^a^	12,360	0.96 (0.92, 1.00)
Switch from uninsured to insured	10,915	**1.09** (1.05, 1.14)
Switch from insured to uninsured	7989	**1.07** (1.02, 1.12)
Always uninsured	27,193	**1.15** (1.10, 1.20)

Instability (6-Category) *p*-value: < 0.001

Note: GEE model adjusted for race, age, comorbidity burden, sex, area-level education, area-level income, site of care and time; **bolded** ORs are significant

^a^Includes switches from private to public, public to private, or among the 3 public groups (Medicare, Medicaid, subsidized)

40,615 patients were included in this analysis (11,624 White, 2911 Hispanic, 23,560 Black and 2520 Asian)

The association between insurance instability and UBP varied significantly by racial group (global *p*-value for interaction of instability and race: *p* = 0.002; Table 3); being always uninsured was not significantly associated with UBP for Hispanics (OR 0.93, ns), Asians (OR 1.02, ns), or whites (OR 1.06, ns) but it was associated with higher rates of UBP for blacks (OR 1.22, CI (1.16, 1.28)). Black patients also had significantly higher rates of UBP if they switched from uninsured to insured, or insured to uninsured, compared to blacks with stable insurance.

**Table 3:** Odds Ratios of Uncontrolled BP for Different Types of Insurance Stability/Instability, Compared to Stable Private Insurance, by Race/Ethnicity, in Two Massachusetts Urban Safety Net Hospital Systems

Adjusted Interaction Model (Instability (6-Category) & Race)								
	Non-Hispanic Whites		Hispanic		Black		Asian	
**Instability (5-Category)**	**n (6-month Intervals)**	**OR (95% CI)*p*-value**	**n (6-month Intervals)**	**OR (95% CI)*p*-value**	**n (6-month Intervals)**	**OR (95% CI)*p*-value**	**n (6-month Intervals)**	**OR (95% CI)*p*-value**
Stable Private (reference)	39,162		3774		49,310		4956	
Stable Public	27,249	**0.85** (0.81, .90)	6957	**0.85** (0.75, .97)	69,537	0.97 (0.93, 1.01)	9399	0.93 (0.82, 1.05)
Switch without losing insurance^a^	2861	0.93 (0.86, 1.01)	590	0.95 (0.79, 1.13)	8080	0.98 (0.93, 1.03)	829	0.96 (0.80, 1.14)
Switch from uninsured to insured	1782	**1.15** (1.04, 1.27)	649	1.01 (0.85, 1.20)	7892	**1.1** (1.05, 1.16)	592	1.13 (0.93, 1.37)
Switch from insured to uninsured	1228	1.05 (0.94, 1.18)	440	1.06 (0.86, 1.31)	5977	**1.09** (1.03, 1.16)	344	0.96 (0.75, 1.23)
Always uninsured	4264	1.06 (0.97, 1.16)	2084	0.93 (0.79, 1.09)	19,266	**1.22** (1.16, 1.28)	1579	1.02 (0.86, 1.22)

Instability (6-Category) *p*-value: < 0.001

Race *p*-value: < 0.001

Overall Interaction of Race and Insurance Instability *p*-value: 0.002

Note: GEE model adjusted for age, comorbidity burden, sex, area-level education, area-level income, site of care and time. 40,615 patients were included in this analysis (11,624 Non-Hispanic White, 2911 Hispanic, 23,560 Black and 2520 Asian). Significant ORs are shown in **bold**

^a^ Includes switches from private to public, public to private, or among the 3 public groups (Medicare, Medicaid, subsidized)

Tabulated odds ratios are in comparison to stable private insurance, within race/ethnicity group

## Discussion

We examined patterns of racial/ethnic disparities in uncontrolled blood pressure (UBP) over the time period spanning the implementation of MA health reform, and examined whether insurance instability was associated with BP control, and whether that association varied by race/ethnicity. We anticipated that patients with less insurance instability would have better BP outcomes.

As expected, and as found in prior literature, [13, 18] we identified racial/ethnic differences in UBP, with less BP control among blacks and Hispanics. We found that insurance instability was associated with worse outcomes, in several ways. Compared to those with stable insurance, patients with insurance switches to becoming either insured or uninsured had worse BP outcomes, as did those who were consistently uninsured, especially always uninsured black patients. In contrast, those with stable public insurance had better outcomes, supporting the value of stable insurance coverage for chronic disease monitoring and treatment. None of these varying associations were significant for Asian patients, perhaps due to their smaller numbers in our sample.

Prior studies have demonstrated both better BP outcomes [2, 3] and no effects related to stable or improved insurance coverage [5]. Insurance instability has been associated with worse health or healthcare utilization outcomes, [1, 6] though prior examinations focused on self-reported health [2] or healthcare utilization outcomes, [1] rather than the clinical indices of BP control assessed during routine clinical care used in this study. Our findings are among the first to elucidate racial/ethnic disparities in clinical outcomes related to insurance instability, in the context of health insurance reform.

Insurance instability had differing associations with UBP, depending on race/ethnicity; we observed the most negative associations for blacks who were always uninsured, and protective associations with outcomes among those with stable public insurance for non-Hispanic whites and Hispanics. These findings suggest that stable health insurance alone – though helpful in controlling BP - is insufficient to fully address uncontrolled BP for everyone, especially given that rates of UBP are still high (regardless of insurance instability), overall. These findings echo other work showing that MA health insurance reform alone was not sufficient to ameliorate racial/ethnic differences in healthcare utilization, [19, 20] and studies of stably insured veteran patients using Veterans Affairs healthcare have found similar rates of uncontrolled BP to those observed in this study [21].

Our findings of differing associations between insurance instability and UBP depending on race/ethnicity may also be plausibly due to the differing role of the unmeasured factors across these subgroups. Insurance instability could be influenced by factors that may also affect BP management. For instance, lower family income / limited education / family stressors (unmeasured in our models) may increase the risk of becoming/remaining uninsured and poor BP management. Therefore, our estimates of the association between instability and poor outcomes may not be causal.

### Limitations

Our study has several limitations. First, MA is a unique health insurance environment, given its low proportion of uninsured patients and its strong safety net prior to the most recent health reform. However, its special features, and its adoption of health insurance reform prior to the national insurance expansion (Affordable Care Act), allow examination of the associations of insurance instability with clinical outcomes in this context. Second, we drew on patient data from only two urban tertiary care hospitals and affiliated community health centers, and focused only on primary care received, so generalizability of the findings may be limited. For example, outcomes among users of urgent care or emergency department care are not clear, though blood pressure management is rarely addressed in urgent and emergency settings. However, there was substantial diversity among patients’ race/ethnicity and sociodemographic characteristics, enhancing generalizability of the findings. Third, as insurance information came from the EMR, it was only available if patients were seen. However, we carried forward conservative values from the past, among patients with known continued care, to address this concern. Fourth, our data included no information about processes or quality of care, or patient adherence to recommended therapy, which may have had additional associations with outcomes. Further, our use of census tract data for income and education was not ideal, but this was the only approach we could identify to remedy the lack of such information in the EMR. Our limited data set did not allow us to explicitly address causality or to examine potential explanations for the finding that being uninsured is specifically associated with UBP for Blacks but not for other groups, such as varying individual level socioeconomic status that may have impacted ability to cover medication costs in the absence of insurance coverage. This is a topic for future research.

Achieving BP control requires clinician prescription of appropriate antihypertensive medications, and patient adherence to such medications, as well as other lifestyle changes. Results from this study support the notion that insurance instability is associated with worse BP outcomes, but that this association varied by patients’ race/ethnicity. Given that racial/ethnic disparities in UBP did not decrease over time, these findings also suggest that stable insurance alone is not enough to decrease racial/ethnic disparities in BP outcomes. Indeed, there may have been additional confounding by systematic racial/ethnic differences in unobserved factors (family income; limited education; other aspects of SES, (such as employment status), stressors, life-style, and other unmeasured social determinants) which could help explain both insurance status and access to BP treatment, which might lead to stronger associations between instability and UBP among some groups (blacks) and not among others.

## Conclusions

We identified racial/ethnic differences in UBP, with less BP control among blacks and Hispanics, finding that insurance instability was associated with worse outcomes, in several ways. Our findings suggest that stable health insurance alone is insufficient to fully address uncontrolled BP for everyone, and also suggest that while health policies that enable insurance stability may be beneficial, clinicians and policy makers must do more than ensure stable insurance to fully alleviate racial/ethnic disparities in hypertension outcomes.

## Data Availability

Study data are stored on a secure, password protected network at Tufts Medical Center. The datasets generated and/or analysed during the current study are not publicly available due to patient confidentiality but are available from the corresponding author on reasonable request.

## Abbreviations

UBP: Uncontrolled blood pressure
BP: Blood pressure
MA: Massachusetts
EMR: Electronic medical record
GEE: Generalized estimating equations
JNC7: Joint National Commission
HS: High school
UBP: Uncontrolled blood pressure

## References

[1] Schoen C, DesRoches C (2000). Uninsured and unstably insured: the importance of continuous insurance coverage. Health Serv Res.

[2] Sommers BD, Gawande AA, Baicker K. Health insurance coverage and health - what the recent evidence tells us. N Engl J Med. 2017;377(6):586–93. 10.1056/NEJMsb1706645 28636831

[3] Wilper AP, Woolhandler S, Lasser KE, McCormick D, Bor DH, Himmelstein DU. Hypertension, diabetes, and elevated cholesterol among insured and uninsured U.S. adults. Health Affairs (Project Hope). 2009;28(6):w1151–9. 10.1377/hlthaff.28.6.w1151 19843553

[4] McWilliams JM, Meara E, Zaslavsky AM, Ayanian JZ (2009). Differences in control of cardiovascular disease and diabetes by race, ethnicity, and education: U.S. trends from 1999 to 2006 and effects of medicare coverage. Ann Intern Med.

[5] Baicker K, Taubman SL, Allen HL, Bernstein M, Gruber JH, Newhouse JP (2013). The Oregon experiment--effects of Medicaid on clinical outcomes. N Engl J Med.

[6] Sommers BD, Gourevitch R, Maylone B, Blendon RJ, Epstein AM (2016). Insurance churning rates for low-income adults under health reform: lower than expected but still harmful for many. Health Affairs (Project Hope).

[7] Freeman JD, Kadiyala S, Bell JF, Martin DP (2008). The causal effect of health insurance on utilization and outcomes in adults: a systematic review of US studies. Med Care.

[8] Smedley BD, Stith AY, Nelson AR (2003). Unequal treatment: confronting racial and ethnic disparities in health care.

[9] Kapoor A, Battaglia TA, Isabelle AP, Hanchate AD, Kalish RL, Bak S (2014). The impact of insurance coverage during insurance reform on diagnostic resolution of cancer screening abnormalities. J Health Care Poor Underserved.

[10] Freund KM, LeClair A, Terrin N, Hanchate AD, Price LL, Moreno-Koehler A, Suzukida J, Kher S, Byhoff E, Kressin NR. Racial Differences in Insurance Stability After Health Insurance Reform. Med Care. 2019;57(4):256–61. 10.1097/MLR.0000000000001078 PMC6605092 30807452

[11] Yoon SS, Gu Q, Nwankwo T, Wright JD, Hong Y, Burt V. Trends in blood pressure among adults with hypertension: United States, 2003 to 2012. Hypertension (Dallas, Tex : 1979). 2015;65(1):54–61. 10.1161/HYPERTENSIONAHA.114.04012 PMC11262548 25399687

[12] Centers for Disease Control and Prevention (CDC). Vital signs: awareness and treatment of uncontrolled hypertension among adults--United States, 2003–2010. MMWR Morb Mortal Wkly Rep. 2012;61:703–9. 22951452

[13] Wong MD, Shapiro MF, Boscardin WJ, Ettner SL (2002). Contribution of major diseases to disparities in mortality. N Engl J Med.

[14] Geography TAMUDo. Texas A & M Geoservices 2019 [Available from: https://geoservices.tamu.edu/Services/Geocode/WebService/GeocoderService_V03_01.asmx.

[15] Chobanian AV, Bakris GL, Black HR, Cushman WC, Green LA, Izzo JL (2003). The seventh report of the joint National Committee on prevention, detection, evaluation, and treatment of high blood pressure: the JNC 7 report. JAMA.

[16] Deyo RA, Cherkin DC, Ciol MA (1992). Adapting a clinical comorbidity index for use with ICD-9-CM administrative databases. J Clin Epidemiol.

[17] Hardin JH, Joseph (2003). Generalized estimating equations.

[18] Kressin NR, Orner MB, Manze M, Glickman ME, Berlowitz D (2010). Understanding contributors to racial disparities in blood pressure control. Circ Cardiovasc Qual Outcomes.

[19] McCormick D, Hanchate AD, Lasser KE, Manze MG, Lin M, Chu C (2015). Effect of Massachusetts healthcare reform on racial and ethnic disparities in admissions to hospital for ambulatory care sensitive conditions: retrospective analysis of hospital episode statistics. BMJ.

[20] Lasser KE, Hanchate AD, McCormick D, Manze MG, Chu C, Kressin NR (2014). The effect of Massachusetts health reform on 30 day hospital readmissions: retrospective analysis of hospital episode statistics. BMJ (Clinical research ed).

[21] Kressin NR, Long JA, Glickman ME, Bokhour BG, Orner MB, Clark C (2016). A brief, multifaceted, generic intervention to improve blood pressure control and reduce disparities had little effect. Ethn Dis.

